# *Anystis baccarum*: An Important Generalist Predatory Mite to be Considered in Apple Orchard Pest Management Strategies

**DOI:** 10.3390/insects5030615

**Published:** 2014-07-24

**Authors:** Andrew G. S. Cuthbertson, Bao-Li Qiu, Archie K. Murchie

**Affiliations:** 1 The Food and Environment Research Agency, Sand Hutton, York YO41 1LZ, UK; 2 Department of Entomology, South China Agricultural University, Guangzhou 510640, China; E-Mail: baileyqiu@scau.edu.cn; 3 The Agri-Food and Biosciences Institute, Newforge Lane, Belfast BT9 5PX, UK; E-Mail: archie.murchie@afbini.gov.uk

**Keywords:** apple orchard, *Anystis baccarum*, predatory mite, chemical

## Abstract

The increasing concern over the continued use of pesticides is pressurising apple growers to look for alternatives to chemical pest control. The re-discovery, and subsequent conservation, of the beneficial predatory mite, *Anystis baccarum* (Linnaeus) (Acari: Anystidae), in Bramley apple orchards in Northern Ireland offers a potential alternative control component for incorporation into integrated pest management strategies. *Anystis baccarum* readily feeds upon economically important invertebrate pest species including European fruit tree red spider mite, *Panonychus ulmi* (Koch) (Acari: Tetranychidae) and show a level of compatibility with chemical pesticides. Recent mis-identification by apple growers of this beneficial mite species had resulted in unnecessary pesticide applications being applied within Northern Irish apple orchards. However, dissemination of information to the apple growers and promotion of the benefits this mite offers in apple orchards has helped to conserve its populations. Apple growers, across the United Kingdom, must be encouraged to be aware of *A. baccarum*, and indeed all predatory fauna, within their orchards and seek to conserve populations. In doing so, it will ensure that the British apple market remains an environmentally sustainable production system.

## 1. Introduction

Fruit crops, especially apple (*Malus* × *domestica* Borkh*.* (Rosaceae)), have a relatively high economic value in the fresh market and therefore cosmetic damage and insect infestation must be kept to a minimum. Insect damaged apple fruit is often only accepted for pulp (*i.e.*, fruit juice or apple pie filler). As a result, control of economically important arthropod pest species must be undertaken.

**Figure 1:**
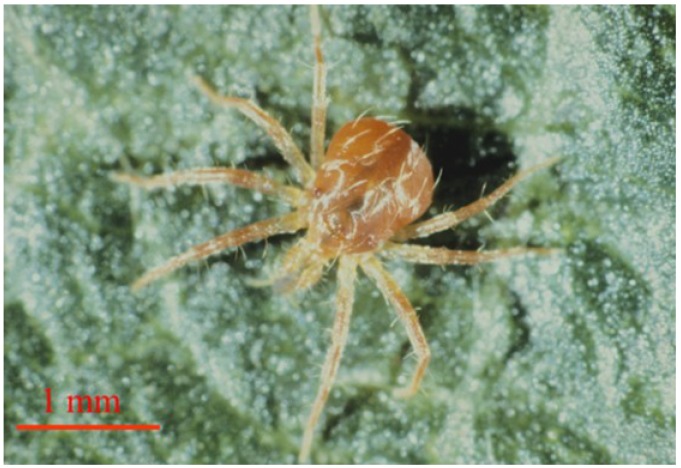
Adult female of the beneficial whirligig mite, *Anystis baccarum* (Photo: Andrew G. S. Cuthbertson^©^).

In non-intensive orchards, phytophagous mite populations can be maintained below economic thresholds by natural acarine predation [1,2]. Much work has been done on the impact of predators, such as *Typhlodromus pyri* Scheuten (Acari: Phytoseiidae) and *Zetzellia mali* (Ewing) (Acari: Stigmaeidae), on populations of both European fruit tree red spider mite, *Panonychus ulmi* (Koch) (Acari: Tetranychidae), and apple rust mite, *Aculus schlechtendali* (Nalepa) (Acari: Eriophyidae) [3,4,5,6,7]. Although these predators are found in Northern Irish Bramley apple orchards, Cuthbertson and Murchie [8] identified the prostigmatid “whirligig” mite, *Anystis baccarum* (Linnaeus) (Acari: Anystidae) (Figure 1), as the most abundant predatory mite in the orchards surveyed. The same study also confirmed that there were fewer *T. pyri* in Northern Irish apple orchards compared to their English counterparts.

**Figure 2:**
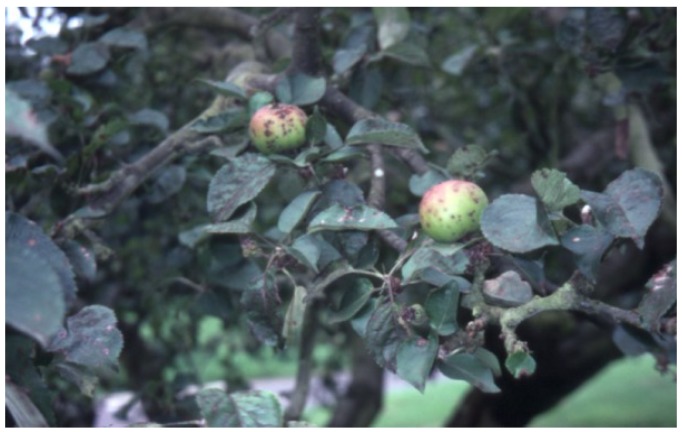
Scab-infected fruit and foliage (Photo: Andrew G. S. Cuthbertson^©^).

In Northern Ireland, the Bramley’s Seedling apple industry is especially important to the rural economy of County Armagh [9]. The annual value of output of the whole apple industry in Northern Ireland is approximately £10 million [9] with Bramley production in County Armagh comprising an estimated 97% of this total [10]. Although apple scab (*Venturia inaequalis* (Cooke) Winter (Ascomycota: Venturiaceae)) (Figure 2) is the largest problem facing apple growers in Northern Ireland [11], invertebrate pest species still pose a significant problem. 

Within the United Kingdom (UK), the Agricultural Development and Advisory Service (ADAS), have set out economic thresholds for integrated production of pome fruits [12]. For the apple pests found in Northern Irish orchards the treatment thresholds used on unspecified cultivars in English and Welsh orchards are shown in Table 1. The spray practice in Northern Ireland’s orchards is probably derived from ADAS recommendations based on the greater occurrence of a wider range of pests in England and Wales. The economic thresholds assume that populations will increase at a certain rate, but this is likely to differ in the cooler climate of Northern Ireland compared to, for example, Southern England. Mowat and Clawson [13] found winter populations of *P. ulmi* to exceed ADAS action thresholds in four Northern Irish orchards out of fifteen sampled. Indeed more recently, Cuthbertson and Murchie [14] conclusively showed that the UK wide ADAS thresholds were simply too low for Northern Irish orchards, with pest populations often vastly exceeding thresholds with no corresponding damage being recorded.

**Table 1:** Agricultural Development and Advisory Service (ADAS) economic thresholds used for the pest species found in Northern Irish apple orchards [12,14].

Sampling unit per tree (visual inspection unless beating specified)	Pest/disease	Threshold per 25 trees	Action if threshold exceeded
			
**Dormant period**			
2 vegetative buds in one year shoots	*Aculus schlechtendali*	Average 10 mites/bud	Treat at mouse ear/green cluster
4 branch nodes on 2-3 year old wood	*Panonychus ulmi*	>30 nodes with >5 eggs	Treat with ovicide pre-blossom
**Bud-burst to mouse ear**			
Two outer rosette leaves	*Aculus schlechtendali*	Average 5 mites/outer leaf	Treat as soon as possible pre-blossom
**Green cluster to pink bud**			
4 trusses	*Rhopalosiphum insertum*	30 trusses infested	}Treat at pink bud
	*Archips podana*	5 trusses infested	
**Late blossom to petal fall**			
2 leaves	*Panonychus ulmi*	Average of 2 mites/leaf	}Treat as soon as possible
	*Aculus schlechtendali*	Average of 5 mites/leaf	
**Fortnightly after petal fall**			
2 leaves	*Panonychus ulmi*	Average 2 mites/leaf	Treat as soon as possible
Pheromone traps	*Archips podana*	>30 moths/trap/week	Treat 7–10 days after threshold catch or immediately if using diflubenzuron

In relation to arthropod pest control in Northern Irish apple orchards, on average two to three sprays of insecticide or acaricide per annum are applied [10]. While this is a small expenditure compared with costs of fungicide application, there are several reasons for the avoidance of unnecessary sprays [15,16]: (1) adverse public attitudes to pesticides have intensified in recent years and this has led to a desire by retailers to reduce dependence on pesticides, especially broad-spectrum neurotoxic compounds that can adversely affect human health or the environment; (2) unnecessary applications of pesticides can foster pest resistance to the chemical seriously compromising efficacy; (3) unnecessary chemical applications are a waste of money in an increasingly competitive business; and (4) within a comparatively stable ecosystem like orchards, the ability of natural enemies to control pests, completely or partly, is well documented. Loss of natural enemies means that minor pests can become more damaging as the natural constraints on their populations have been removed [14].

## 2. Sampling for *Anystis baccarum*

Leaf-dwelling predators, such as the mites *T. pyri* and *Z. mali*, can be sampled, among other collecting methods, by leaf washing [17]. However, larger predatory species are generally more mobile and cannot be sampled in this way as they disperse when vegetation is disturbed. Various trapping devises have been described by several authors for trapping insect fauna including earwigs, mites and mirids [18,19,20]. Using a selection of novel trapping devices, a study within Northern Irish apple orchards determined that *A. baccarum* was the most abundant beneficial species in the orchards [8]. Straw traps proved best for trapping, not only *A. baccarum*, but all predatory mites sampled in the study [8].

## 3. The Biology of *Anystis baccarum*

**Figure 3:**
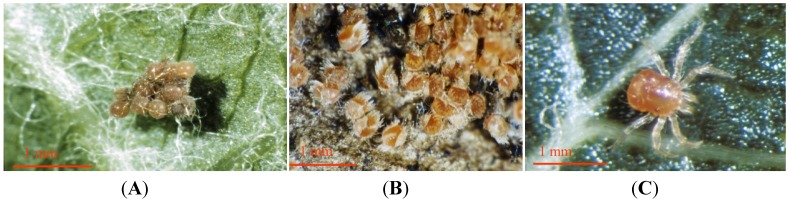
(**A**) Egg batch of *Anystis baccarum*; (**B**) Eggshells splitting and juveniles emerging; (**C**) Six-legged larval stage (Photos: Andrew G. S. Cuthbertson^©^).

This species has no males and reproduction is by parthenogenesis [21]. Maturing eggs can easily be seen through the body sclera of the female and, before oviposition, the eggs occupy the entire body of the female [22]. Female mites lay on average two egg clusters comprising 22 eggs per cluster [23]. They will readily lay eggs on moist surfaces (for example, on moist tissue paper in the laboratory) [23]. These are interwoven with transparent filaments (Figure 3a) [24]. Eggs are laid both on the bark of the tree trunks [25] and also in soil litter around the base of the trees [22,23]. Hatching eggs can be recognised by the splitting of the eggshell and the emergence of the legs of the pre-larva (Figure 3b) [24]. The pre-larvae are small (0.2 mm diam.) six legged and immobile. They do not feed and scarcely leave the split eggshells, existing only on egg yolk stored in the intestine [22]. However, after 10–15 days the pre-larvae become active larvae (Figure 3c) and migrate into the crowns of trees where they feed on suitable prey [22,23]. The larval stage lasts five to six days and is followed by three nymphal instars which are eight-legged (Figure 1), taking in total about one month to develop into adult form. Adults then live for 15–17 days [22] and can be as large as 1.5 mm in diameter. 

All life stages of *A. baccarum* are cannibalistic [26] which makes it difficult to rear them in the laboratory, although Golovach [27] concluded it to be possible but not easy. Newly-hatched larvae may feed on eggs or pre-larvae in the same tube [28]. All other life stages are preyed on by active adult mites. However, in the field it is difficult to assess the importance of this behavior as cannibalism may not occur to the same extent. Cannibalistic behavior has been stated to be an important factor in the dispersal of *A. baccarum* or could be of survival value where the eggs or pre-larvae form part of the diet of larvae hatching in the winter [26].

**Figure 4:**
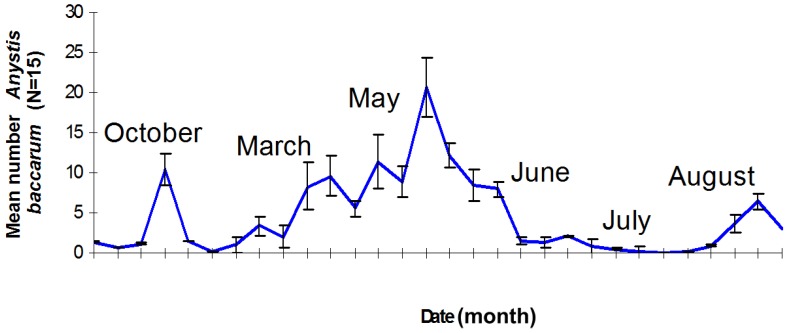
The phenology of *Anystis baccarum* in a Northern Irish apple orchard [23].

*Anystis baccarum* occurs within Northern Irish orchards all year round (Figure 4), with abundance peaking during spring and early summer [23]. At least three generations were observed in the orchards [23], similar to field observations in Guangzhou, China [29].

## 4. Identification of *Anystis baccarum*

**Figure 5:**
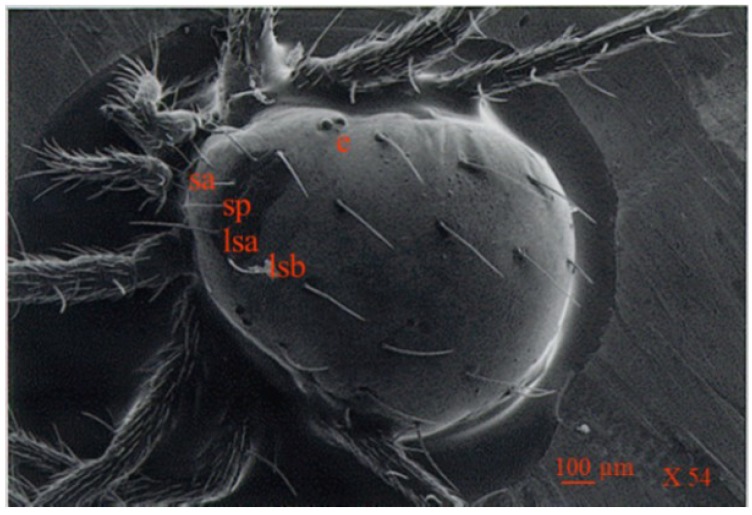
Scanning electron micrograph showing dorsal view of *Anystis baccarum*. Anterior margin bears a pair of sensilla (sa); prodorsal shield bears two pairs of long setae (lsa + lsb) and a pair of sensilla (sp); two pairs of eyes are located postero-lateral to prodorsal shield (e) (Photo: Andrew G. S. Cuthbertson^©^).

**Figure 6:**
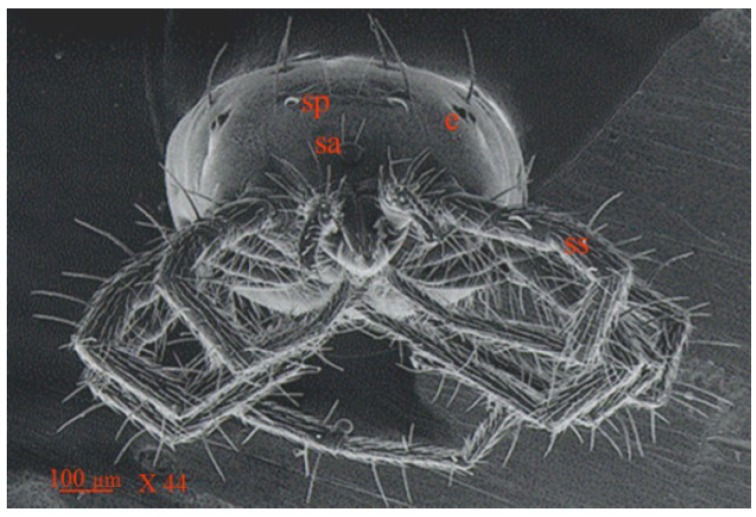
Scanning electron micrograph showing anterior view of *Anystis baccarum*. Legs densely covered in short smooth setae (ss); anterior margin of idiosoma bearing sensilla (sa); prodorsal shield bearing pair of sensilla (sp); eyes located postero-lateral to prodorsal shield (e) (Photo: Andrew G. S. Cuthbertson^©^).

**Figure 7:**
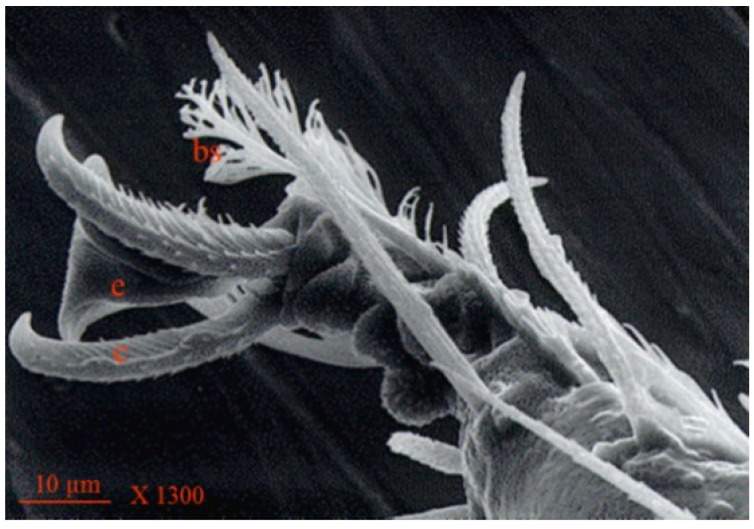
Scanning electron micrograph of *Anystis baccarum* claw. Each tarsus terminates in two claws (c) and an empodium (e); two brush like setae present at base of claws (bs) (Photo: Andrew G. S. Cuthbertson^©^).

**Figure 8:**
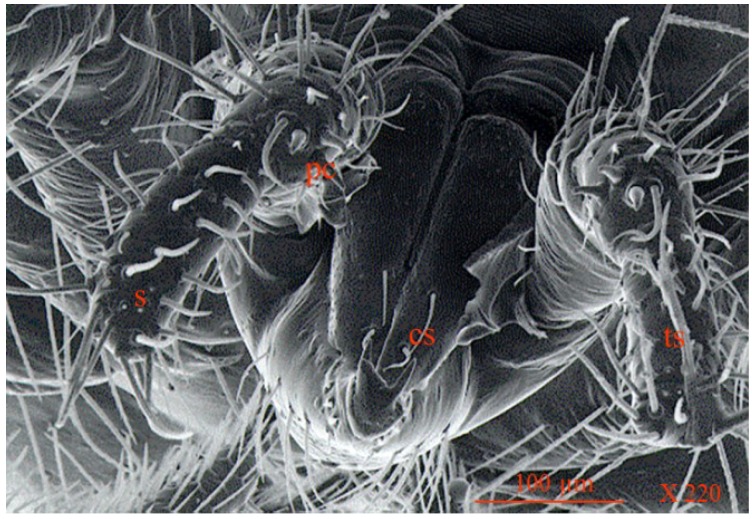
Scanning electron micrograph of gnathosoma region of *Anystis baccarum*. Palptibia bears three claws (pc); palptarsus bears four small solenidia (s) and many long serrated setae of which the terminal setae is the longest (ts); the chelicerae each bear two setae (cs) (Photo: Andrew G. S. Cuthbertson^©^).

**Figure 9:**
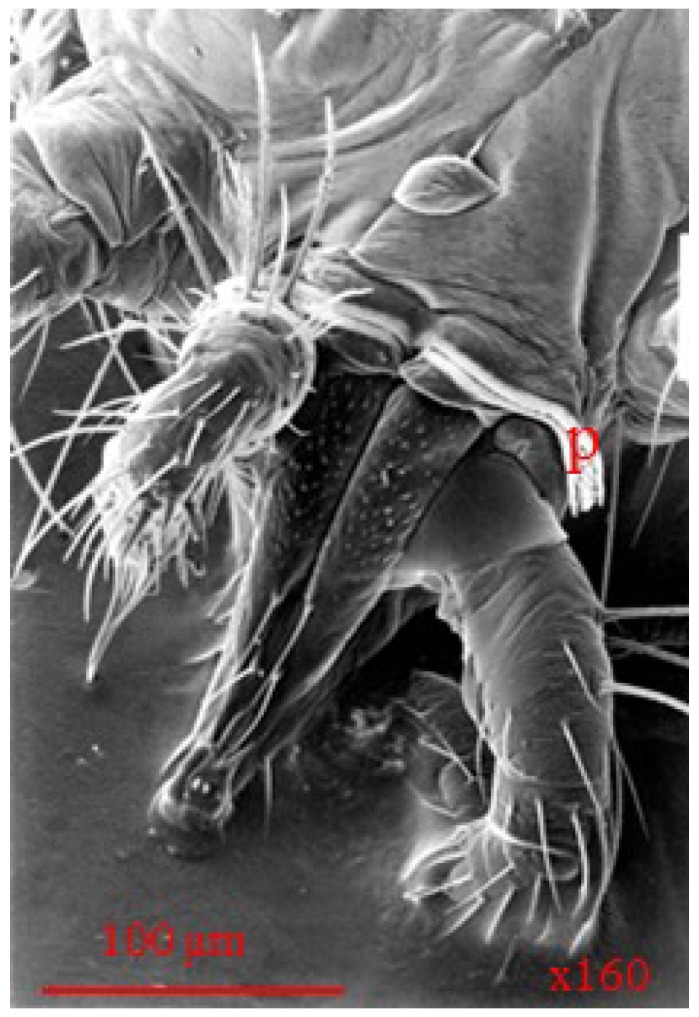
Scanning electron micrograph showing the distal half of the reticulated peritremes (p) flared on *Anystis baccarum* (Photo: Andrew G. S. Cuthbertson^©^).

Certain characteristics are listed by Meyer and Ueckermann [21] from which *A. baccarum* may be identified:

(1) Dorsum—the prodorsal shield is almost rounded anteriorly and indented posteriorly, bearing two pairs of long setae and a pair of sensilla. The anterior margin of the idiosoma has also a pair of sensilla. Additionally, two pairs of eyes are located postero-lateral to the prodorsal shield (Figure 5);
(2) Legs—the legs of *A. baccarum* are densely covered with short smooth setae (Figure 6). Each tarsus terminates in two claws and an empodium with brush like setae present (Figure 7);
(3) Gnathosoma—the palptibia has three claws and the palptarsus bears four small solenidia. There are many long serrated setae of which the terminal setae is the longest. The two chelicerae each contain two setae (Figure 8). The distal half of the reticulated peritremes are flared (Figure 9).

**Figure 10:**
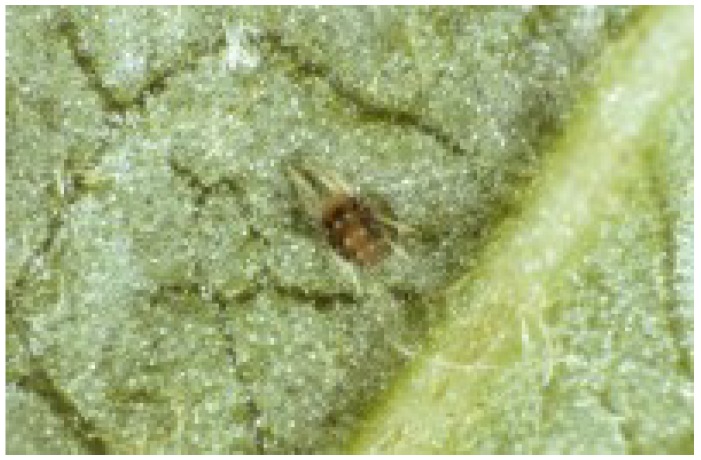
The European fruit tree red spider mite, *Panonychus ulmi* (Photo: Andrew G. S. Cuthbertson^©^).

*Anystis baccarum* adults however, can be easily spotted on the fruit and foliage of apple trees as they are red/orange in color, long-legged, and very mobile. The common name of “Whirligig mite” derives from the mite’s rapid, whirling movement—akin to a whirligig spinning toy. The situation among Northern Irish apple growers was that, until recently, they were unaware of the presence of this mite within their orchards. Therefore, they were assuming that any red mite on the apple trees was the pest species, *P. ulmi* (Figure 10) [30,31]. However, certain characteristics enable *A. baccarum* adults to be easily identified in the field [15]:

(1) *Anystis baccarum* ranges in size from 1.0 to 1.5 mm in diameter. This is a lot larger than *P. ulmi*, which when fully grown is about 0.4 mm in diameter;
(2) *Anystis baccarum* moves rapidly over the branches and foliage of the trees, whereas, *P. ulmi* is relatively sedentary and only found on the under-side of leaves;
(3) Eggs of *P. ulmi* are visible on the twigs of the trees during the winter months, whereas *A. baccarum* lays eggs under loose bark on the trunk or in the soil surrounding the tree base.

To help overcome the problem of mis-identification of this beneficial mite and to ensure the elimination of unnecessary pesticide sprays, *A. baccarum* identification cards [32] were designed and distributed around Northern Irish apple growers during the 2009 growing season. These were gratefully received and the information uptake by the local apple growers was very positive. As a result, over the preceding years, several fruit growers have now avoided the need to apply chemical sprays within their orchards against what has now been confirmed as a beneficial species [33].

## 5. Potential of *Anystis baccarum* to Control Invertebrate Pests

**Figure 11:**
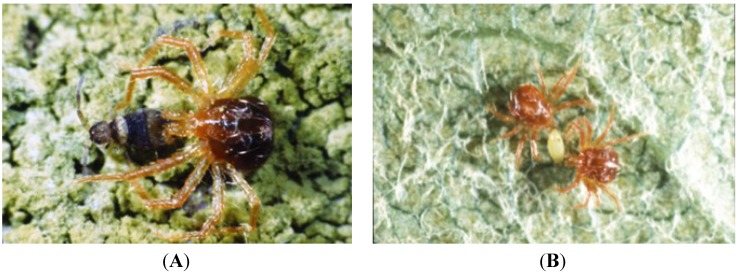
*Anystis baccarum* attacking (**A**) Collembola and (**B**) apple-grass aphid prey (Photos: Andrew G. S. Cuthbertson^©^).

Mites of the genus *Anystis* have been suggested as bio-control agents of pest arthropods [34] as they have been observed feeding on a variety of prey species throughout the world (Figure 11) (Table 2). In the UK, *A. baccarum* can become abundant during times of aphid infestation in cereal fields [35], whereas in New Zealand, *A. baccarum* plays an important role in the predation of tortricid larvae in apple orchards [36]. *Anystis baccarum* was also found to increase in number during outbreaks of fruit tree red spider mite in Canadian apple orchards [37] and offer control of phytophagous mites in orchards and blackcurrant plantations in Russia [22,38,39]. Much work in China has also proven the predatory potential of *A. baccarum* on pests including the tea leafhopper [40], longan psyllid [41] and the tea red spider mite [42]. As *A. baccarum* has been known elsewhere to feed on pest species (Table 2) that also occur in British apple orchards [43], this predatory mite will no doubt be an important component of pest management strategies to be employed in the British Isles.

**Table 2:** References to *Anystis baccarum* feeding on invertebrate pest species.

Prey	Crop/Host	Country	Source
Phytophagous mites including *Panonychus ulmi* (Fruit tree red spider mite)^1,2^	Apple orchards, Black-currant plantations	Russia, Northern Ireland, China, England, Canada	[6,22,23,29,34,36,37,38]
*Sitobion avenae*, *Rhopalosiphum insertum* (Aphids)^1^	Cereals, Apple trees	England, Northern Ireland, China	[23,26,29,35]
*Empoasca vitis* (Tea leaf hopper)	Tea plants	China	[40]
*Cornegenapsylla sinica* (Longan psyllis)^2^	Orchards	China	[41]
*Cydia pomonella, Archips podana* (Tortricid larvae)^1,2^	Apple trees	New Zealand, Northern Ireland	[36,43]
*Erythroneura* spp. (Leafhoppers)	Grape vineyards	U.S.A	[44]
*Trioza erytreae* (Citrus psylla)	Citrus	South Africa	[45]
*Monelliopsis pecanis* (Yellow pecan aphid)^1^	Pecans	South Africa	[45]
*Limothrips cerealium*, *Thrips flavus* (Thrips)	Cereal fields, flowering plants	England	[35,46]
*Oulema melanopa* (Cereal leaf beetle)	Cereal crops	Sweden	[47]
*Cacopsylla* spp. (Psyllid eggs)	Sugar beet	Czechoslovakia	[48]
*Matsucoccus matsumurae* (Pine blast scale)	Pine trees	China	[49]
*Oligonychus coffeae*, *Tetranychus kanzawai* (Tea mites)^1,2^	Tea plants	China, Korea	[42,50,51]
*Aculus schlechtendali* (Apple rust mite)^1,2^	Apple trees	Northern Ireland	[52]

Pests which are (or are closely related to) Bramley orchard pest species^1^. *Anystis baccarum* considered to be a bio-control agent^2^.

Studies undertaken in the laboratory to look at the potential of *A. baccarum* as a bio-control agent against orchard invertebrate pest species have indicated that *A. baccarum* readily feeds upon apple pests in the laboratory, including: *P. ulmi*, *A. schlechtendali*, *Rhopalosiphum insertum* (Walker) (Hemiptera: Aphididae) and *Bryobia rubrioculus* Scheuten (Acari: Tetranychidae) [23]. The former three species are listed as being of economic importance within Northern Irish orchards [13]. Field studies have also proven the worth of *A. baccarum* in controlling pest species [41,42]. As *A. baccarum* feeds on tortricid moth larvae in New Zealand [36], it will therefore presumably also feed upon *Archips podana* (Scopoli) (Lepidoptera: Tortricidae) and *Cydia pomonella* (Linnaeus) (Lepidoptera: Tortricidae) larva within UK orchards [43].

## 6. Compatibility of *Anystis baccarum* with Chemicals

**Figure 12:**
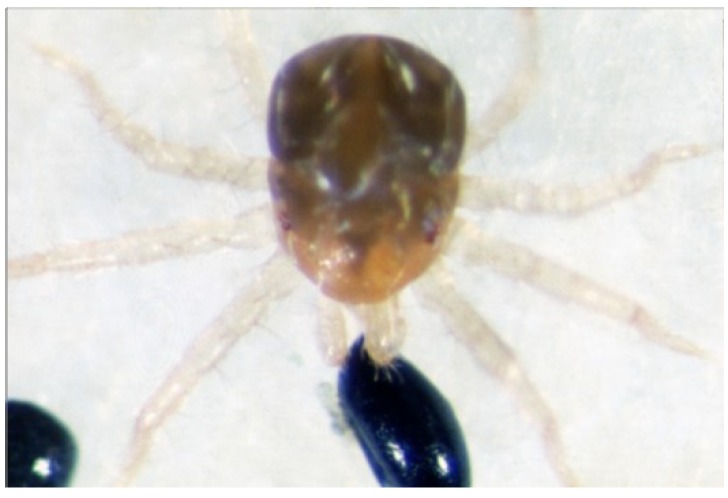
*Anystis baccarum* feeding on an overwintering *Rhopalosiphum insertum* egg (Photo: Andrew G. S. Cuthbertson^©^).

Until recently the only information on the impact of chemical pesticides or fungicides on *A. baccarum* was from a study in Russia in 1974 [53]. Within the British Isles, Cuthbertson and Murchie [54] determined that *A. baccarum* had the potential of being compatible with various chemical fungicides (e.g., dithianon) commonly used for apple scab control. This same study also highlighted a strong link between leaf quality and apple rust mite numbers. Cuthbertson and Murchie [55] also determined that orchard winter-washes and chemical pesticide applications aimed at controlling invertebrate pests had detrimental effects on *A. baccarum* populations. The beneficial mite was removed by the chemicals and as a result of this, and presumably depletion of other natural enemies, pest populations, such as, *A. schlechtendali*, increased within the orchards [14,55]. However, both *A. schlechtendali* [56] and also *R. insertum* [57,58] may have a beneficial role within the orchards in that they are a valuable food source for sustaining over-wintering populations of *A. baccarum* (Figure 12).

In Canada, work by Laurin and Bostanian [59,60] proved that residues of the fungicides sulphur, captan and myclobutanil were harmless to *A. baccarum* as were the insecticides methoxyfenozide, acetamiprid, imidacloprid and spinosad. Cuthbertson and Murchie [61] found pirimicarb and tebufenpyrad to have a low toxicity to *A. baccarum* while phosalone proved very toxic to the mite. Studies in China determined that azadirachtin and imidacloprid had a weak toxicity against *A. baccarum* [40] while a mixture of abamectin and fenpropathrin proved to be very toxic to *A. baccarum* [51]. *Anystis baccarum* therefore offers much potential to be incorporated into current integrated pest management (IPM) strategies within British orchards for arthropod pest control [62].

## 7. Conclusions

In the development of orchard IPM programs, generalist predatory mites, such as *A. baccarum*, must also be fully researched to determine their impact upon pest species and included within any such IPM system implemented. Horticultural advisors and fruit growers alike must be fully aware of the biodiversity that occurs within an orchard ecosystem and ensure the correct identification of pest and beneficial species, thus eliminating unnecessary chemical applications [32]. The inclusion of generalist predatory species within pest control programs has the potential to lead to a more sustainable apple production system, not only within Northern Ireland, but the British Isles as a whole and elsewhere [62,63].
